# Impact of stent strut link location in proximal balloon edge dilation technique for bifurcation percutaneous coronary intervention

**DOI:** 10.1007/s12928-023-00981-4

**Published:** 2024-01-31

**Authors:** Teruyoshi Kume, Takeshi Nishi, Yoshinobu Murasato, Satoshi Koto, Yoshitaka Sasahira, Hiroshi Okamoto, Ryotaro Yamada, Terumasa Koyama, Tomoko Tamada, Koichiro Imai, Yoji Neishi, Shiro Uemura

**Affiliations:** 1 Department of Cardiology, Kawasaki Medical School, Kurashi, Japan; 2 Department of Cardiology and Clinical Research Institute, National Hospital Organization Kyushu Medical Center, Fukuoka, Japan

**Keywords:** Bifurcation, Coronary, Stent, Optical coherence tomography

## Abstract

The single-stent strategy has generally been accepted as the default approach to bifurcation percutaneous coronary intervention. We have proposed the proximal balloon edge dilation (PBED) technique to prevent stent deformation during side branch (SB) dilation. This bench study aimed to evaluate the impact of stent link location and stent design on stent deformation, obstruction by stent struts at a jailed SB ostium, and incomplete stent apposition in the proximal optimization technique (POT)-PBED procedure. A coronary bifurcation model was used. We intentionally set the absence or presence of stent link on the carina (link-free or link-connect) under videoscope observation and compared stent parameters between 3- and 2-link stents (*n* = 5 each, *n* = 20 total). In the link-free group, the SB jailing rate of 3-link stents was significantly higher than that of 2-link stents (15.5 ± 5.1% vs. 6.6 ± 1.2%, *p* = 0.009). In the link-connect group, the SB jailing rate of 3-link stents was significantly lower than that of 2-link stents (30.0 ± 4.5% vs. 39.0 ± 2.6%, *p* = 0.009). In the bifurcation segment, the rate of incomplete stent apposition was significantly lower for 3-link stents of the link-connect group than for 2-link stents of the link-connect group (3.3 ± 4.2% vs. 19.0 ± 7.8%, *p* = 0.009). For both stent designs, ellipticity ratio was higher for link-connect group than link-free group. Link location as well as stent cell design greatly impacted stent deformation during the POT-PBED procedure.

**Graphical abstract:**

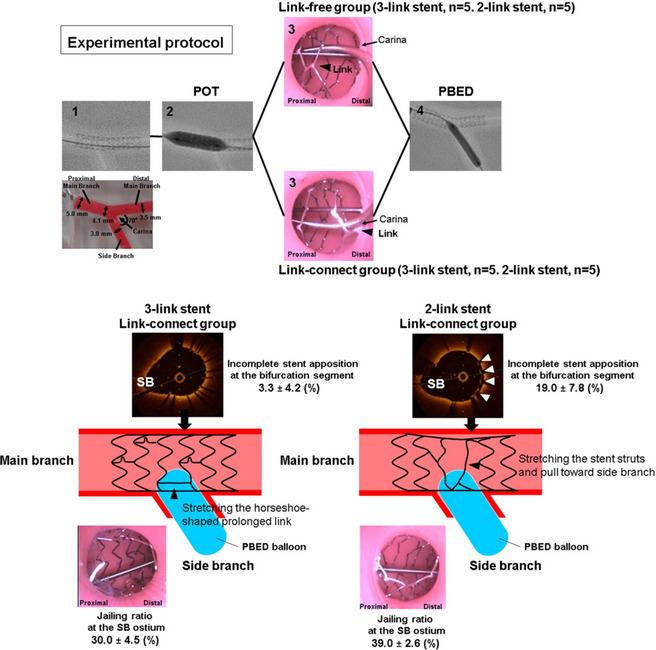

## Introduction

Bifurcation percutaneous coronary intervention (PCI) remains an area of ongoing procedural challenge [1–3]. Simple strategies with a cross-over single-stent implantation or provisional side branch (SB) stenting have generally been accepted as default approaches for bifurcation PCI [4–7]. We previously proposed the proximal balloon edge dilation (PBED) technique to prevent stent deformation during SB dilation, and achieved excellent results in bench tests and in clinical cases [8–11]. Clinical 3D optical coherence tomography (OCT) studies have shown that absence of a stent link at the SB ostium immediately after main branch (MB) stent implantation was associated with a lower frequency of obstruction of stent struts at a jailed SB ostium and stent strut malapposition (incomplete stent apposition) after kissing balloon inflation (KBI) [12–14]. However, it remains unclear whether the presence of stent links on the carina (link-free group or link-connect group) correlates to stent deformation or to incomplete stent apposition and obstruction of stent struts at a jailed SB ostium following the proximal optimization technique (POT)-PBED procedure. In addition, it remains unclear whether 2-link or 3-link stent design provides better results with the POT-PBED procedure in bifurcation PCI. Therefore, this bench study aimed to evaluate the impact of stent link location and stent design on stent deformation, obstruction by stent struts at a jailed SB ostium, and incomplete stent apposition in the POT-PBED procedure.

## Methods

### Experimental protocols

**Fig. 1:**
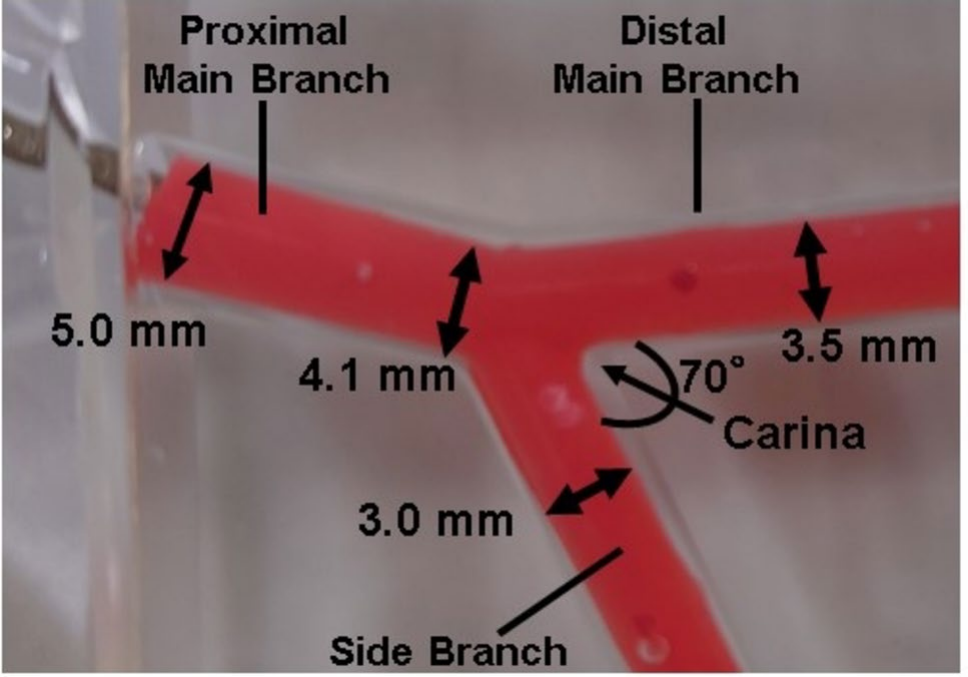
Coronary bifurcation bench model. A flexible urethane coronary bifurcation bench model with 1-mm thickness was custom made with lumen diameters of 3.5 mm for the main branch and 3.0 mm for the side branch, and carina angle of 70 degrees

**Fig. 2:**
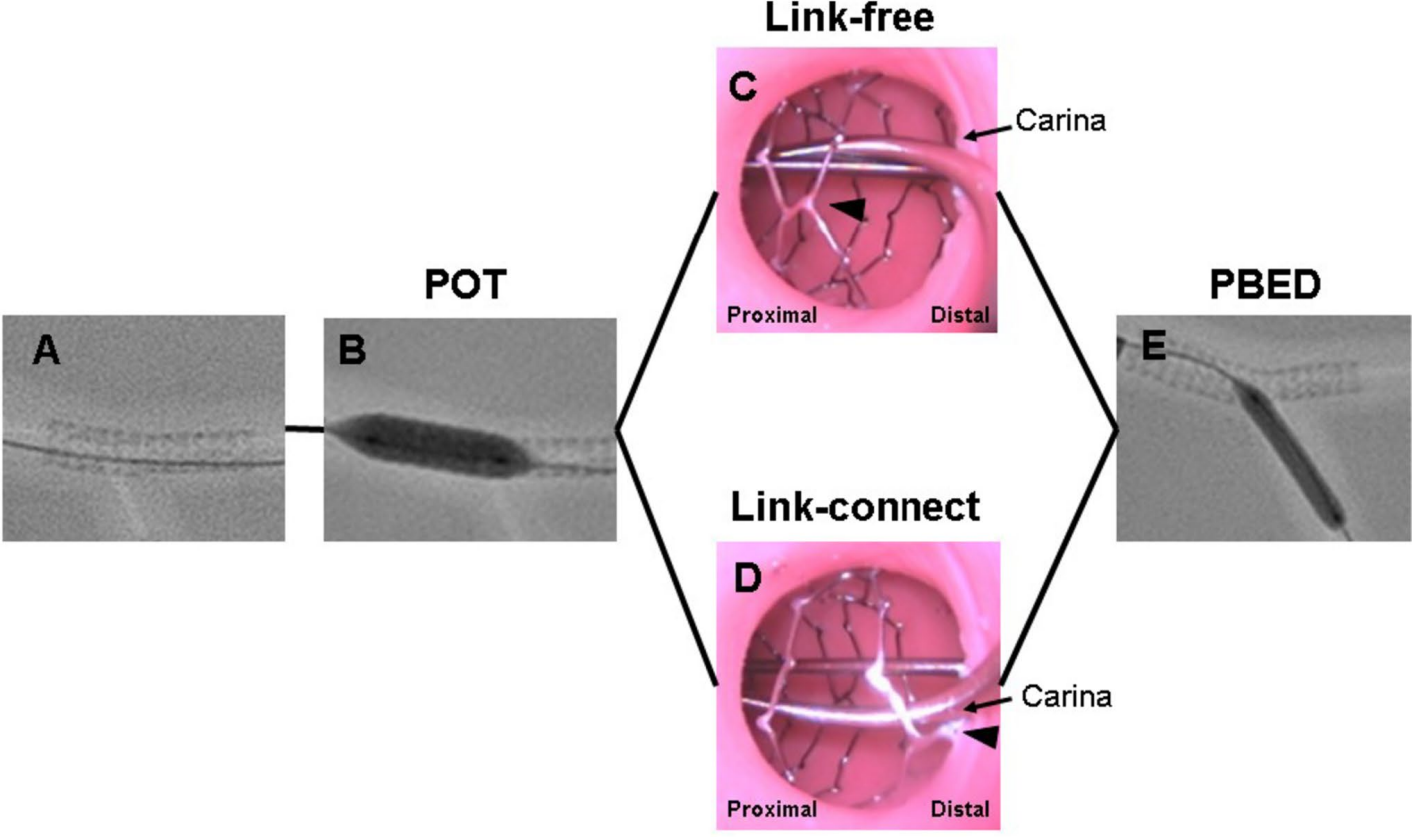
Flow chart of the protocols. Initially, 3.5-mm stents were implanted in the main branch (**A**). A proximal optimization technique (POT) was then performed using a 4.5-mm non-compliant balloon (**B**). To assess the impact of link location, we intentionally set the absence or presence of stent link on the carina under videoscope observation (C: link-free group, D: link-connect group) (link: arrowhead). The side branch ostium strut cells were opened by a 3.0-mm non-compliant balloon at the nominal pressure (12 atm) for 10 s using the proximal balloon edge dilation (PBED) technique

A fractal coronary bifurcation bench model made of flexible urethane (thickness, 1 mm) was specially designed with lumen diameters of 3.5 mm for the distal main branch and 3.0 mm for the side branch, with carina angle of 70° (Cross Medical Service Co., Tokyo, Japan) (Fig. 1). An experimental bench test was performed using each of the following 3- and 2-link coronary stents as the main branch stent in the coronary bifurcation model: XIENCE Sierra 3.5 × 23 mm (*n* = 10) (Abbott Vascular, Santa Clara, CA) and Synergy 3.5 × 24–32 mm (*n* = 10) (Boston Scientific, Marlborough, MA). Stents were deployed as follows. First, 3.5-mm stents were implanted in the MB in accordance with compliance charts from the manufacturer to reach a diameter of 3.70 mm three times for 10 s. We intentionally set the absence or presence of stent link on the carina (link-free or link-connect) under videoscope observation and compared the impact of link location between 3- and 2-link stents (Fig. 2). Second, POT was performed using a 4.5-mm non-compliant balloon at the nominal pressure for 10 s. The POT balloon was positioned precisely, with the medial edge of the distal radiopaque marker lying in the cross-sectional plane of the carina. Third, another guidewire was advanced into the SB with the distal cell crossing under direct visual observation using a videoscope (IPLEX TX; Olympus, Tokyo, Japan) because distal cell rewiring at the SB ostium has been reported to minimize stent deformation and obstruction by stent struts at jailed SB ostium after SB balloon dilation [12, 13]. Fourth, the SB ostium strut cells were opened by a 3.0-mm non-compliant balloon at the nominal pressure (12 atm) for 10 s using the PBED technique described previously. [8–11]

### Optical coherence tomography (OCT) and videoscope analyses

**Fig. 3:**
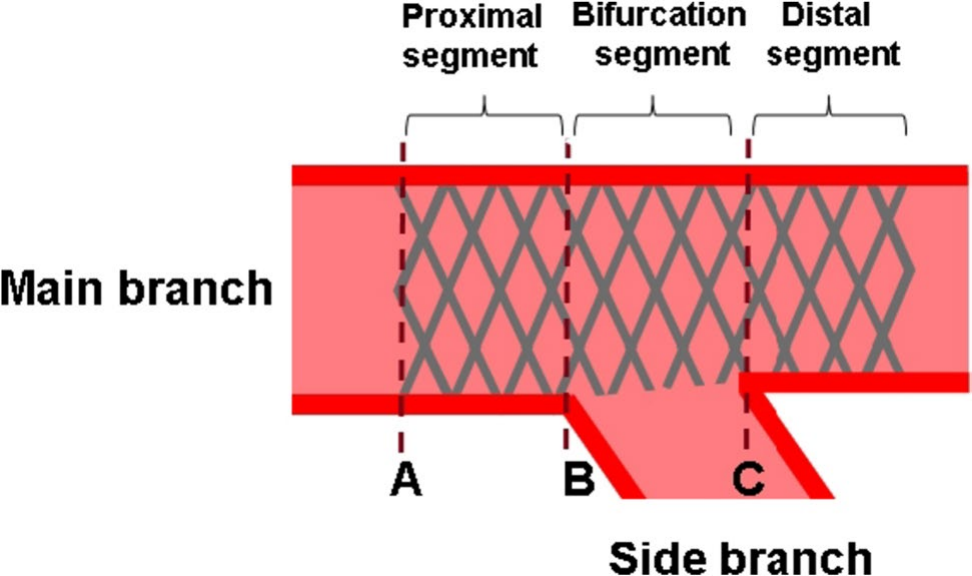
Schema of a longitudinal reconstruction OCT image. Stent area was measured at the proximal (**A**) and distal (**B**) edges of the proximal MB segment and at the proximal edge of the distal MB segment (**C**)

OCT images were obtained using an FD-OCT system (Dragonfly OPTIS and OPTIS stent optimization software; Abbott Vascular, St. Paul, MN). The FD-OCT catheter was placed distal (> 10 mm) to the stented lesion and pulled back to the proximal edge of the stent using a motorized catheter pullback system (18 mm/s). OCT recording was performed after SB ostial treatments and OCT analysis was performed millimeter by millimeter. Ellipticity ratios at the proximal and distal MBs were calculated as maximal diameter/minimal diameter, with 1.0 corresponding to perfect circularity. Maximal value of the ellipticity ratio was adopted. Malapposed strut on OCT was defined as a distance of > 150 µm between the center reflection of the strut and the vessel wall on axial-section OCT [15]. Overall malapposed struts except for those at the SB ostium were quantified on each axial section as the percentage of malapposed struts to the total number of struts analyzed (rate of incomplete stent apposition). The stent area of the proximal and distal edges at the proximal MB segment and the proximal edge at the distal MB segment were measured (Fig. 3A–C).

The videoscope was inserted into the SB to obtain images of the SB ostium. Obstruction by stent struts at a jailed SB ostium was evaluated as the SB jailing ratio, calculated as (A1/A2)$\times$ 100 (%), where A1 is the total area jailed by stent struts, and A2 is the area of the ostium. Both A1 and A2 were measured manually on digital planimetry using ImageJ version 1.50i software (National Institutes of Health, Bethesda, MD).

After SB ostial treatments, stent parameters such as ellipticity ratio and rate of incomplete stent apposition by OCT were assessed in the proximal segment, bifurcation segment, and distal segment and compared among the following four groups: 3-link stents of link-free group, 3-link stents of link-connect group, 2-link stents of link-free group, and 2-link stents of link-connect group. The SB jailing ratio obtained from videoscopy was assessed and compared among the four groups.

### Statistical analysis

All analyses were performed using JMP version 14.2.0 software (SAS Institute, Cary, NC). Continuous variables are presented as the mean ± standard deviation. Wilcoxon’s nonparametric statistical test for continuous quantitative variables was used for comparison among the groups. Values of *p* < 0.05 were considered significant.

## Results

**Fig. 4:**
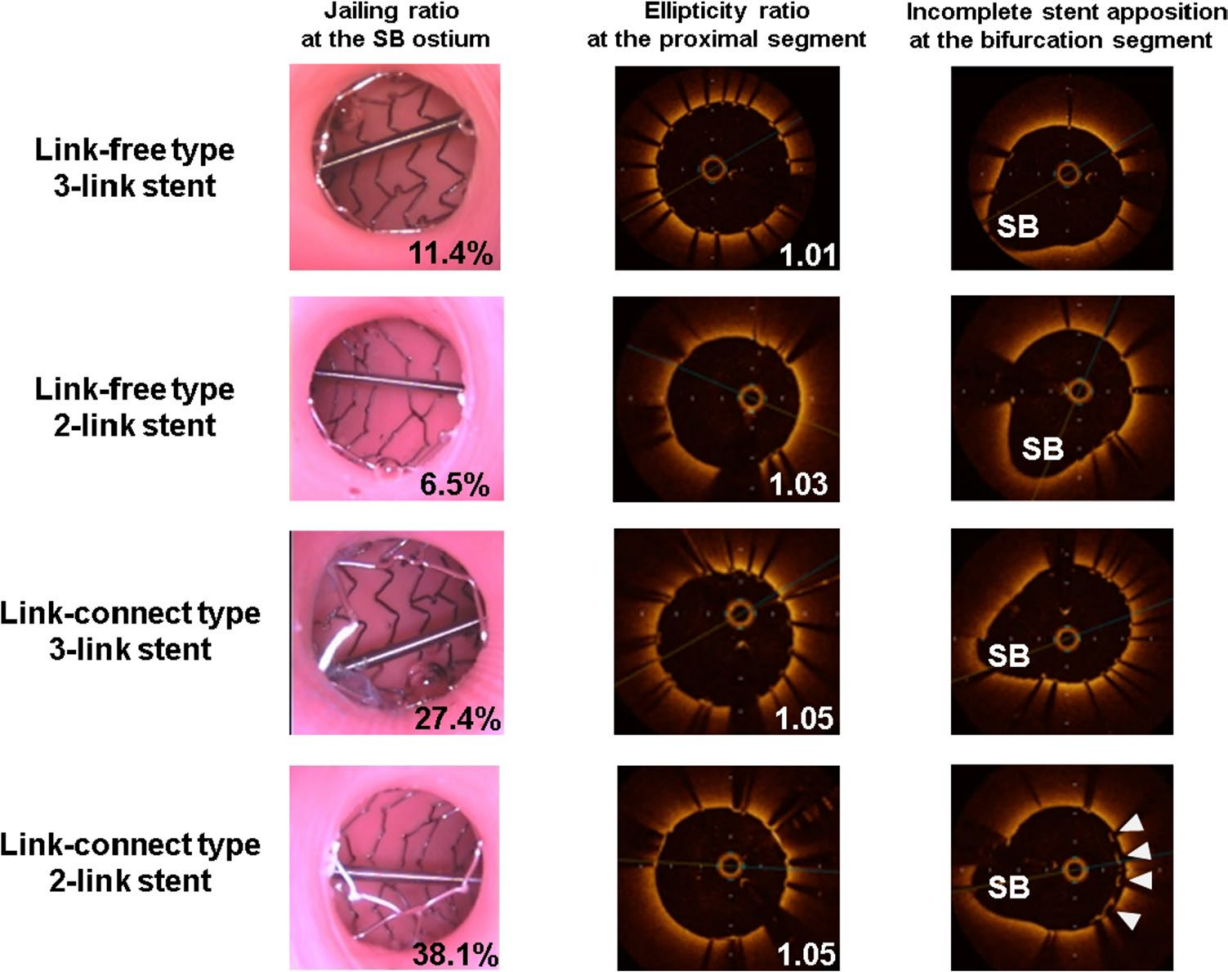
Representative videoscope images of side branch ostium and OCT images at the proximal main branch and bifurcation segment after PBED procedure. In the link-free group, the SB jailing rate of 3-link stents was higher than that of 2-link stents (11.4%, 6.5%) (left). In the link-connect group, the SB jailing rate of 3-link stents was lower than that of 2-link stents (27.4%, 38.1%). The ellipticity ratio at the proximal main branch segment was similar between the 3-link and 2-link stents. For both stent designs, the ellipticity ratio was higher for link-connect group than link-free group (1.05, 1.05, 1.01, and 1.03, respectively) (middle). After proximal balloon edge dilation (PBED), incomplete stent apposition at the bifurcation segment was observed in 2-link stents of the link-connect group (right, arrowhead). SB: side branch

Table 1 lists the stent parameters after the POT-PBED procedure. In the link-free group, the SB jailing rate of 3-link stents was significantly higher than that of 2-link stents. On the other hand, in the link-connect group, the SB jailing rate of 3-link stents was significantly lower than that of 2-link stents. Both stent designs showed a higher SB jailing rate for link-connect group than link-free group (3-link stent: 30.0 ± 4.5% vs. 15.5 ± 5.1%, *p* = 0.009, and 2-link stent: 39.0 ± 2.6% vs. 6.6 ± 1.2%, *p* = 0.009, respectively). Rates of incomplete stent apposition at proximal and distal segments did not differ significantly between the two groups. In the bifurcation segment, the rate of incomplete stent apposition was significantly lower for 3-link stents of link-connect group than for 2-link stents of link-connect group. As for stent expansion, stent areas of the proximal and distal edges at the proximal MB segment and proximal edge at the distal MB segment were similar between 3-link and 2-link stents. For both stent designs, stent area of the proximal edge at the distal MB segment was significantly lower for link-connect group than link-free group (3-link stent: 9.7 ± 0.6 mm^2^ vs. 10.5 ± 0.3 mm^2^, *p* = 0.018, and 2-link stent: 9.4 ± 0.9 mm^2^ vs. 10.8 ± 0.4 mm^2^, *p* = 0.028, respectively). The ellipticity ratio at the proximal and distal segments was similar between 3-link and 2-link stents. For both stent designs, ellipticity ratio both at the proximal and distal segments was significantly higher for link-connect group than link-free group (at the proximal segment, 3-link stent: 1.06 ± 0.03 vs. 1.02 ± 0.02, *p* = 0.047, and 2-link stent: 1.05 ± 0.02 vs. 1.03 ± 0.01, *p* = 0.009, at the distal segment, 3-link stent: 1.09 ± 0.01 vs. 1.04 ± 0.03, *p* = 0.021, and 2-link stent: 1.13 ± 0.08 vs. 1.05 ± 0.02, *p* = 0.036, respectively). Figure 4 shows representative SB ostial videoscope images and OCT images in the different groups.

**Table 1:** Comparison of stent parameters following the POT-PBED procedure

	Link-free group		*P* value	Link-connecting group		*P* value
	3-link stent (*n* = 5)	2-link stent (*n* = 5)		3-link stent (*n* = 5)	2-link stent (*n* = 5)	
Jailing rate, (%)	15.5 ± 5.1*	6.6 ± 1.2^†^	0.009	30.0 ± 4.5	39.0 ± 2.6	0.009
Incomplete stent apposition, (%)						
Proximal segment	0.0 ± 0.0	0.0 ± 0.0	1.000	1.4 ± 1.8	0.5 ± 1.1	0.290
Bifurcation segment	5.1 ± 9.7	14.3 ± 9.9	0.160	3.3 ± 4.2	19.0 ± 7.8	0.009
Distal segment	3.4 ± 1.7	1.8 ± 1.2	0.076	2.6 ± 3.0	1.0 ± 0.9	0.332
Stent area (mm^2^)						
Proximal edge at the proximal MB segment	15.6 ± 0.3	15.4 ± 0.2	0.190	15.4 ± 0.4	15.4 ± 0.4	0.752
Distal edge at the proximal MB segment	15.0 ± 0.9	14.6 ± 0.3	0.833	14.1 ± 0.4	14.7 ± 0.6	0.172
Proximal edge at the distal MB segment	10.5 ± 0.3*	10.8 ± 0.4^†^	0.283	9.7 ± 0.6	9.4 ± 0.9	0.602
Ellipticity ratio						
Proximal segment	1.02 ± 0.02*	1.03 ± 0.01^†^	0.246	1.06 ± 0.03	1.05 ± 0.02	0.754
Distal segment	1.04 ± 0.03*	1.05 ± 0.02^†^	0.754	1.09 ± 0.01	1.13 ± 0.08	0.465

Values are mean ± SD

*POT* proximal optimization technique, *PBED* proximal balloon edge dilation

*3-link stent of link-free group vs. 3-link stent of link-connecting group. *p* value < 0.05

^†^2-link stent of link-free group vs. 2-link stent of link-connecting group. *p* value < 0.05

**Fig. 5:**
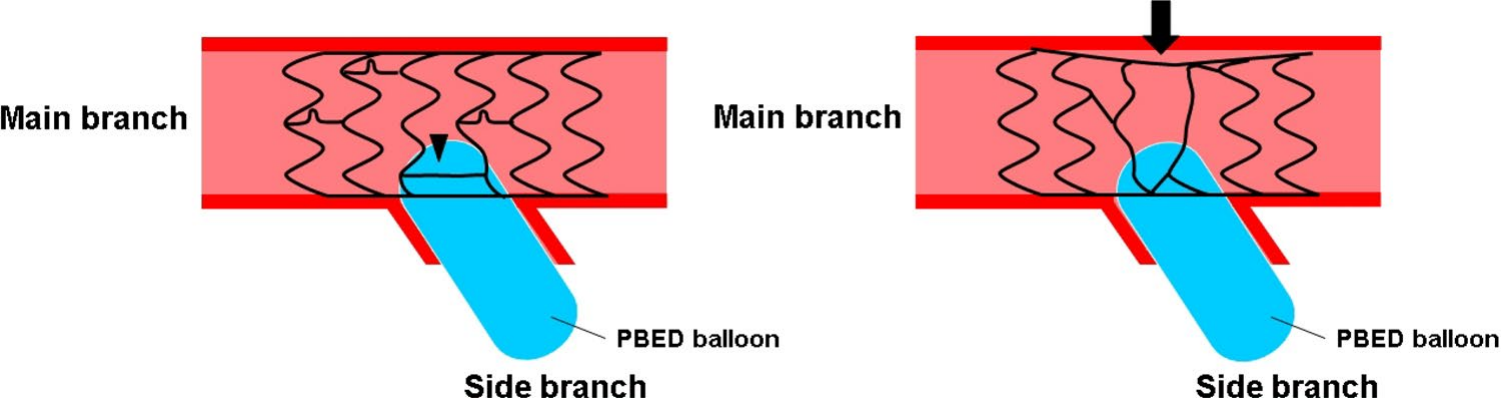
Stent configuration in the link-connect group during side branch dilation between the 3-link stent and the 2-link stent. In the link-connect group, the XIENCE stent, the 3-link stents used in the present study might minimize the deformation of stent cells by stretching the horseshoe-shaped prolonged link during side branch (SB) dilation (arrowhead, left). On the other hand, the Synergy stent which characterized as the 2-link stents with peak to valley design might be deformed toward the SB and result in an incomplete stent apposition of the bifurcation segment during SB dilation (arrow, right)

## Discussion

The present experimental bifurcation bench model study evaluated two different stents with two different link locations and showed that the link-connect group might induce stent deformation resulting in higher SB jailing, ellipticity ratios and lower stent area of the proximal edge at the distal MB segment following the POT-PBED procedure. The rate of incomplete stent apposition of the bifurcation segment in the 2-link stents was numerically higher than that of 3-link stents of the link-free group, and was statistically higher than that of the 3-link stents of the link-connect group.

Implantation of an MB stent across the SB ostium followed by POT is recommended as a single-stent strategy [4]. Various types of SB dilation treatment are available after the POT procedure, including KBI and single balloon dilation techniques. To the best of our knowledge, this is the first report to clarify the effect of stent link location on stent deformation after the POT-PBED procedure according to stent cell design. Link location at the SB ostium (link-free or link-connect) is an important factor influencing stent deformation in bifurcation PCI [12, 13, 16]. Thus, it was thought that stents with a 2-link cell design would be preferable for bifurcation PCI. The present results showed that in the link-free group, the SB jailing rate of 2-link stents was significantly better than that of 3-link stents; whereas in the link-connect group, the SB jailing rate was significantly worse than in the link-free group. These data are consistent with those of previous clinical studies that found that the presence of a stent link on the carina immediately after MB stent implantation (link-connect group) was associated with high SB jailing rates after KBI [12, 13]. In particular, in the link-connect group, the SB jailing rate of 2-link stents was significantly worse than that of 3-link stents. Furthermore, 2-link stents might be easily deformed toward the SB as a result of SB dilation, which explains the significantly higher rate of incomplete stent apposition of the bifurcation segment after PBED procedure in the link-connect group. The XIENCE stent, the 3-link stents used in the present study has a horseshoe**-**shaped prolonged link connecting each stent strut. This characteristic might minimize the deformation of stent cells by stretching the horseshoe-shaped prolonged link during the POT-PBED procedure. If the stent links on the carina (link-connect) was present, it would be expected the improvement of the SB jailing rate with high pressure or multiple SB dilation. However, the increase of the rate of incomplete stent apposition at the bifurcation segment would be also expected (see Fig. 5). To avoid the influence of link location, KBI with high-pressure SB dilation might be preferable especially for 2-link stents of the link-connect group. The present results indicate that in addition to stent cell design, link location as assessed by 3-dimensional OCT is also important; and that selection of the most appropriate stent design and the method of SB dilation is necessary to optimize bifurcation PCI.

Clinical impacts of jailed struts on the SB ostium have been reported. Recently, we reported that the presence of a stent link on the SB ostium was related to SB narrowing in the chronic phase. [17] More recently, fenestrated restenosis developed from the jailed struts across the SB ostium was introduced in the 15th consensus document from the European bifurcation club [4]. These data suggest that obstruction of stent struts at a jailed SB ostium is important for SB flow disturbance due to delayed neointimal coverage at follow-up. In addition, stent struts at a jailed SB ostium have been recognized to pose certain risks for late or very late stent thrombosis [18–21]. Therefore, the presence of stent links on the carina (link-free group or link-connect pattern) that relates the obstruction of stent struts at a jailed SB ostium might be clinically important.

As for the type of stent, two different types of stents were examined in the present study. The Synergy stent characterized as the 2-link stents with peak to valley design might be deformed toward the SB and result in an incomplete stent apposition of the bifurcation segment during SB dilation. Even though the latest Ultimaster stent (TERUMO, Tokyo, Japan) is characterized as the same 2-link stents with peak to peak design, the number of stent crowns increased from 8 (Ultimaster Tansei™, TERUMO, Tokyo, Japan) to 10 (Ultimaster Nagomi™ LV, TERUMO, Tokyo, Japan), which may prevent stent deformation during SB dilation by stretching stent struts. Future large-scale investigations are necessary to elucidate the exact impact of stent design and link location on stent behavior during the POT-PBED procedure.

### Limitations

Several limitations to this study need to be considered. First, the bench model can never entirely represent in vivo coronary anatomies and cannot exactly reproduce real-life conditions. Second, the present experimental study used two types of coronary stent. Since every stent has a different stent cell design besides 2-link or 3-link stents, further bench tests are needed that compare a greater variety of coronary stent types. Third, the limited sample size may have influenced the conclusions. Fourth, considering the stent strut thickness and OCT image resolution, a malapposed strut on OCT was defined as a distance of > 150 µm between the center reflection of the strut and the vessel wall in the present study [15]. A previous clinical study demonstrated that malapposed stent struts with a distance of < 355 µm between the center reflection of the strut and the vessel wall immediately after everolimus-eluting stent implantation had resolved spontaneously at 8–12-month follow-up [22]. In the present study, the maximum distance between the center reflection of the strut and the vessel wall was < 400 µm in all cases. Therefore, the clinical impact of the small malapposed struts of the bifurcation segment that were observed in the present study is unclear, and future clinical studies are needed. Finally, in the present study, coronary bifurcation bench model with bifurcation angle of 70° assuming left main bifurcation was used with reference to a consensus document for bench testing and coronary artery bifurcations from the European Bifurcation Club [23]. A wide bifurcation angle would be associated with higher rate of incomplete stent apposition at the bifurcation segment after SB inflation [9]. Further studies are required to verify our observations using coronary bifurcation bench model with different bifurcation angle.

## Conclusions

Link location as well as stent cell design strongly affected stent deformation during the POT-PBED procedure. Two-link stents of the link-connect group had the highest SB jailing rate and rate of incomplete stent apposition at the bifurcation segment.

## Data Availability

The data that support the findings of this study are available from the corresponding author, (T.K.), upon reasonable request.
